# The Effectiveness of Intraosseous Basivertebral Nerve Ablation in the Treatment of Nonradiating Vertebrogenic Pain: A Systematic Review

**DOI:** 10.7759/cureus.37114

**Published:** 2023-04-04

**Authors:** Marcellina Nwosu, Walter Y Agyeman, Aakash Bisht, Ankit Gopinath, Ameer Haider Cheema, Keyur Chaludiya, Maham Khalid, Ann Kashmer Yu

**Affiliations:** 1 Internal Medicine, California Institute of Behavioral Neurosciences & Psychology, Fairfield, USA; 2 Interventional Pain Management and Primary Care, El Paso Pain Center, EL Paso, USA; 3 Internal Medicine, Piedmont Athens Regional Medical Center, Athens, USA; 4 Medicine, Government Medical College, Amritsar, IND; 5 Internal Medicine, Kasturba Medical College, Manipal, IND; 6 Internal Medicine, Doctors at Doorstep, Surat, IND

**Keywords:** nonradiating discogenic low back pain, nonradicular low back pain, basivertebral nerve rhizotomy, vertebrogenic low back pain, vertebrogenic pain, chronic low back pain, axial low back pain, basivertebral nerve neurotomy, basivertebral nerve ablation, basivertebral nerve

## Abstract

Intraosseous basivertebral nerve ablation has shown sustained efficacy in treating chronic axial low back pain (LBP) in patients with type 1 or 2 Modic changes. This systematic review aims to determine the efficacy of intraosseous basivertebral nerve radiofrequency ablation in treating nonradiating axial chronic LBP compared to standard therapy, sham, or without contrast. The population of interest is individuals greater than or equal to 18 years old with chronic nonradiating vertebrogenic pain. The key outcome was the percentage of patients with greater than or equal to 50% pain reduction, greater than or equal to 10-point improvement in function and disability measured by the Oswestry Disability Index (ODI), greater than or equal to two-point pain reduction in the visual analog scale (VAS) or numerical pain rating scale, and a decrease in opioid utilization by 10 morphine milligram equivalents.

Three databases, PubMed, MEDLINE, and Google Scholar, were used to retrieve the studies for the review. Two independent reviewers assessed the studies for inclusion using the validated tools for quality appraisal. There were 286 articles in total; however, only 11 publications with extensive data on 413 participants matched the inclusion criteria and were used for this review. At three months, a majority of the participants reported greater than or equal to 10-point improvement in the ODI, a measure of functional and disability improvement on a 10-point scale, and greater than or equal to two-point improvement in the VAS. A good number of patients in the basivertebral nerve ablation (BVNA) arm reported complete pain resolution demonstrating therapy success and the superiority of BVNA over sham and standard treatment.

Basivertebral nerve radiofrequency ablation, among other criteria, is a safe and minimally invasive therapy that significantly lowers pain and impairment in individuals with vertebrogenic pain with distinct Modic type 1 and 2 changes at lumbar vertebra three-sacral vertebra one (L3-S1) vertebral levels. Proper patient selection and exact procedural methods are essential to the success of basivertebral nerve neurotomy. The findings of the existing investigations require confirmation by nonindustry-funded, large-scale, high-quality trials using generalizable study participants.

## Introduction and background

Chronic low back pain (CLBP) or vertebrogenic pain is one of the debilitating conditions frequently encountered in pain management. CLBP is a low back pain (LBP) lasting more than six months. It is one of the most common causes of disability, absenteeism from work, and excessive consumption of healthcare resources worldwide [1-11]. The annual cost of LBP in the United States is estimated to be more than $100 billion [12]. It is the leading cause of chronic opioid use resulting in opioid misuse or overdose in patients on greater than 90 morphine milligram equivalent a day (MME) [8]. Because of the cost and health effects of the opioid epidemic, the National Academy of Medicine recommended the intervention procedures approach for managing chronic back pain [8]. Surgical fusion has provided modest pain relief but has not eliminated the chronic use of opioids in these patients. Since 1990, the rate of LBP has been spiraling due to the increasing number of the aging population [13]. According to statistics, 577.0 million people were living with CLBP in the world in 2017 compared to 377.5 million in 1990, with a lifetime prevalence of 60% to 80% [1,2,12,14]. While some people will recover from LBP, a minority of this population will proceed to debilitating CLBP resulting in a high socioeconomic burden.

About 30% of the population in the United States has LBP, with approximately 50 million physician visits yearly [6]. In the United States, an estimated 30 million adults with LBP progress to CLBP [6]. According to the 2010 Global Disease Burden, LBP is about 60-70% in the United States and Europe [15]. LBP is estimated to have a yearly economic impact on the United States economy of $100 billion, or two percent of gross domestic product, in the form of lost productivity and incapacity [7,12,15]. Recently, there has been a noted increase in the incidence of LBP among people between the ages of 35-55 years. Despite the high prevalence of LBP, the etiology remains nonspecific and can range from genetic causes to low educational status, smoking, obesity, advancing age, and psychosocial triggers such as depression and excessive stress [7,15]. Due to the lack of a standardized diagnostic reference for LBP, 85% of patients are diagnosed with nonspecific LBP, necessitating expensive therapies, long-term opioid use, and ultimately spinal fusion surgery with a 20% reoperation rate in two to four years [4,9-11].

Pathophysiology

CLBP is multifactorial and can be discogenic or axial, radiating or nonradiating, and specific, or nonspecific. Despite the innovations and progress in medical diagnosis, about 80% to 90% of LBP are nonspecific, resulting in a lack of clarity in treatment modalities with an associated mixed outcome. This is because of the multidimensional nature of LBP and the complexity of pinpointing the pain generator [14]. The treatment complexities for LBP are due to the multiple anatomical structures contributing to symptoms of LBP. These include the lumbosacral facet and sacroiliac joints, as well as the spinal nerve roots, ligaments, and intervertebral discs. Recognizing these specific etiologies permits targeted interventions to optimize clinical outcomes [16]. Different interventional pain procedures target these anatomical pain generators [10].

CLBP can result from nociceptive signals originating from spinal and paraspinal structures. The annulus fibrosis, the outer layer, and the nucleus pulposus, the inner layer, are the two separate layers that make up the intervertebral disc. The hypersensitive nociceptors of the degenerating intervertebral disc, which originate from the annulus fibrosis, were once thought to be responsible for transmitting pain (annulogenic pain). Despite this, current research has demonstrated that the vertebral body endplate contributes significantly to the pathologic innervation linked to disc degeneration and transmits pain signals via the basivertebral nerve (BVN) (vertebrogenic pain) [1,2,4,17,18].

The endplate comprises cartilage and bone, separating the intervertebral disc from the adjoining vertebrae [19]. The cartilage aspect of the endplate contains chondrocytes scattered throughout the extracellular matrix. The bony element of the end plate is similar to the vertebral cortex and has properties that resemble a thickened, porous layer of fused trabecular bone. The bony component of the endplate contains hematopoietic cells, nerves, fat cells, and sinusoids that perfuse the intervertebral disc. The endplate is innervated by the BVN giving out fibers that enter the vertebra via the posterior basivertebral foramen [4,18,19]. BVN bundle is a branch of the sinuvertebral nerves that enters the vertebral body through the central vascular foramen and innervates the vertebral endplates [7]. According to the immunohistochemical analysis, BVN contains a high density of nerve fibers that are rich in nociceptors and inflammatory mediators such as substance P, calcitonin gene-related peptide, and protein gene product 9.5, corroborating the nociceptive role of BVN in the conveyance of vertebrogenic pain signals in CLBP [1-5,7,12,20]. The endplates are vulnerable to calcification and damage due to excessive load from activities of daily living, physiological aging, and strains from disc degeneration [16,17]. The damaged endplates cause increased communication between the nucleus pulposus and bone marrow leading to the release of inflammatory mediators and chronic inflammation, notable as Modic changes (MCs) in magnetic resonance imaging (MRI) [1,2,16,17,21]. 

The pathophysiology of the Modic endplate alterations that cause vertebrogenic discomfort has undergone significant advancement. Current evidence has shown that cytokines play a role in the development of MCs. Degenerating disc contiguous to MCs produces high levels of cytokines and osteoclastic factors, which control the composition of adjoining bone marrow and change bone mass [20]. Toll-like receptors activated by damaged-associated molecular patterns also play a role in MCs. Bone marrow lesions seen in osteoarthritis demonstrate many similar characteristics to MCs. Additionally, male gender, high serum lipids, age, and obesity also increase bone marrow lesions and, in turn, explain the pathophysiology of MCs [20].

Modic modifications can be of three types: vertebral endplate disruption, fissuring, degeneration, and active inflammation are all examples of type 1 MCs [9-11]. They manifest as hypodense in the longitudinal relaxation time (T1)-weighted (T1W) MRI sequence and hyperintense in the transverse relaxation time (T2)-weighted (T2W) MRI sequence [9-11]. Type 2 MCs are fatty infiltration of the bone marrow, which appear hyperintense in both T1 and T2 MRI sequences. Type 3 MCs appear hypodense in both T1 and T2 MRI sequences. The increased communication between the vertebral bone marrow from disc and endplate degeneration results in the release of inflammatory mediators. The central nervous system interprets the inflammatory responses received by BVN as LBP [3,4,5,17]. At the endplates, the sensitivity of the nerves increases with increased bone damage.

Moreover, the density of BVN in damaged endplates is higher when compared to nondamaged endplates confirming the link between BVN and CLBP. Patients with CLBP associated with MCs present with increased frequency and duration of LBP and worse outcomes with conservative therapies and after discectomy. This group of patients is more likely to seek medical care, unlike patients with LBP without MCs [3,4,5,17,20]. 

Diagnosis and management

The diagnosis and management of vertebrogenic pain are challenging. Due to the high rate of false positives, provocative discography, which was once utilized to diagnose discogenic pain, is today debatable. The increase in LBP that patients experience following discography may be explained by the knowledge that discography promotes endplate deflection, which amplifies the release of nociceptors and causes pain [3,4,5]. MRI is the gold standard for diagnosing vertebrogenic pain secondary to MCs [1,2,3,4,5,17,20], in addition to information gathered during a history and physical examination.

Current evidence shows that basivertebral nerve ablation (BVNA), a minimally invasive procedure, is safe and effective in managing CLBP in patients with type 1 and type 2 MCs on MRI [20]. A recent clinical trial that evaluated the effectiveness of BVNA demonstrated that BVNA is superior to surgical sham in CLBP management [8]. BVN ablation targets BVNs adjacent to the central vertebral body and ablates them, leading to interruption in pain transmission [3,4,5].

Objectives and rationale

This systematic review aimed to examine the most recent research on the safety, efficacy, adverse effects, and complications of BVN radiofrequency ablation in managing vertebrogenic pain in patients with MCs. Numerous research studies have been published about the effectiveness of BVN radiofrequency ablation, which the Food and Drug Administration (FDA) has approved for treating vertebrogenic discomfort with type 1 and type 2 MCs at lumbar vertebra three (L3) through sacral vertebra one (S1) vertebral levels [22]. 

## Review

Method

The identification, selection, evaluation, and synthesis of the studies used in this systematic review were conducted in accordance with the Preferred Reporting Items for Systematic Reviews and Meta-Analyses (PRISMA) 2020 guidelines to ensure transparency, accuracy, and comprehensiveness of the review [23]. Specific inclusion and exclusion criteria were established for this systematic review to target the population and intervention of interest. The population were adults aged 18 years and older with nonradiating CLBP and MRI evidence of Modic type 1 and type 2 changes. The intervention of interest was intraosseous basivertebral nerve radiofrequency ablation (BVN RFA). 

The primary outcome of interest was greater than or equal to (≥) 50% pain reduction in the visual analog scale (VAS) or numerical pain rating scale (NRS). The secondary outcomes include ≥10-point improvement in function in the Oswestry disability index (ODI), greater than or equal to two-point pain reduction in the NRS, mean reduction in the NRS, VAS, and ODI, and decreased or discontinuation of opioid intake, and improvement in the quality of life. There is no restriction on the duration of the studies and the outcomes. The studies included for review were prospective randomized controlled trials, including double-blinded, single-blinded, open-label, single-arm trials, narrative reviews, systematic reviews, and meta-analyses. Reports, letters to editors, book chapters, case studies, expert opinions, studies conducted outside the United States and Canada, studies written in languages other than English, manuscripts, studies with quality assessment less than 70% on validated scales, and studies older than five years were excluded. The clinical studies that evaluated the effectiveness of intraosseous BVN rhizotomy for the treatment of CLBP in patients with evidence of MCs on the MRI were obtained by searching PubMed using the medical subject headings (MeSH) strategy, MEDLINE, and Google Scholar. The searches were completed on June 23, 2022. Some studies were extracted from the cited references of the included publications. The search strategies and results are shown in Table 1.

**Table 1:** Search Strategy and Results MeSH: Medical Subject Headings

Databases Searched	Date of Search	Keywords/Concepts Used	Search Result	Filters
PubMed MeSH Strategy	June 23, 2022	(("Basivertebral"[All Fields] AND ("nerve"[All Fields] OR "nerve s"[All Fields] OR "nerved"[All Fields] OR "nerves"[All Fields]) AND ("ablate"[All Fields] OR "ablated"[All Fields] OR "ablates"[All Fields] OR "ablating"[All Fields] OR "ablation"[All Fields] OR "ablational"[All Fields] OR "ablations"[All Fields])) OR ("Basivertebral"[All Fields] AND ("nerve"[All Fields] OR "nerve s"[All Fields] OR "nerved"[All Fields] OR "nerves"[All Fields])) OR ("radiofrequency ablation"[MeSH Terms] OR ("radiofrequency"[All Fields] AND "ablation"[All Fields]) OR "radiofrequency ablation"[All Fields]) OR ("neuralgia/drug therapy"[MeSH Major Topic] OR "neuralgia/epidemiology"[MeSH Major Topic] OR "neuralgia/pathology"[MeSH Major Topic] OR "neuralgia/physiopathology"[MeSH Major Topic] OR "neuralgia/prevention and control"[MeSH Major Topic] OR "neuralgia/therapy"[MeSH Major Topic])) AND (("Non-radiating"[All Fields] AND "Discogenic"[All Fields] AND ("pain"[MeSH Terms] OR "pain"[All Fields])) OR (("chronic"[All Fields] OR "chronical"[All Fields] OR "chronically"[All Fields] OR "chronicities"[All Fields] OR "chronicity"[All Fields] OR "chronicization"[All Fields] OR "chronics"[All Fields]) AND ("low back pain"[MeSH Terms] OR ("low"[All Fields] AND "back"[All Fields] AND "pain"[All Fields]) OR "low back pain"[All Fields])) OR ("low back pain"[MeSH Terms] OR ("low"[All Fields] AND "back"[All Fields] AND "pain"[All Fields]) OR "low back pain"[All Fields]) OR (("vertebrogenic"[All Fields] OR "vertebrogenous"[All Fields]) AND ("pain"[MeSH Terms] OR "pain"[All Fields])) OR ("Discogenic"[All Fields] AND ("back pain"[MeSH Terms] OR ("back"[All Fields] AND "pain"[All Fields]) OR "back pain"[All Fields])) OR (("chronic"[All Fields] OR "chronical"[All Fields] OR "chronically"[All Fields] OR "chronicities"[All Fields] OR "chronicity"[All Fields] OR "chronicization"[All Fields] OR "chronics"[All Fields]) AND "Discogenic"[All Fields] AND ("pain"[MeSH Terms] OR "pain"[All Fields])) OR ("Modic"[All Fields] AND "Type"[All Fields] AND ("change"[All Fields] OR "changed"[All Fields] OR "changes"[All Fields] OR "changing"[All Fields] OR "changings"[All Fields])) OR ("low back pain/drug therapy"[MeSH Major Topic] OR "low back pain/epidemiology"[MeSH Major Topic] OR "low back pain/physiopathology"[MeSH Major Topic] OR "low back pain/therapy"[MeSH Major Topic]))	254	Articles Published within the Last Five Years
MEDLINE	June 23, 2022	Basivertebral nerve AND Basivertebral nerve ablation AND Discogenic pain AND Ablation AND Vertebrogenic back pain	3	Studies within the Last Five Years
Google Scholar	June 23, 2022	Basivertebral nerve ablation AND spinal nerve AND nonradiating AND discogenic pain AND vertebrogenic pain	29	Articles Published from 2010 - 2022

Study selection

The publications were independently evaluated for inclusion by two authors with formal training in evidence-based medicine and quality research using the validated quality appraisal tools. Studies are included for review if they satisfy the following criteria: studies with relevant information on intraosseous BVN neurotomy for the treatment of CLBP with MCs, those conducted with appropriate methodology and procedural technique, those that analyzed and reported data using authenticated metrics and tools, and those that clearly reported the outcome of interest, risks, and complications related to the treatment. 

Data items and collection

The following information was taken from the studies: bibliographic data such as the authors and year of publication, study designs, the setting in which the studies were conducted, participant selection methods, descriptions of the procedures and techniques, and other procedural reports, as well as details about author disclosures such as study funding and conflicts of interest, outcomes measures for pain reduction, and improvement in disability and function.

Assessment of risk of bias and methodology of the study

In order to ensure that the participants in the studies were representative of the target population, a rigorous methodologic evaluation was conducted to evaluate the quality of the research using validated techniques and the patient selection process. Other factors evaluated include using validated tools for outcome measurement, use of a control group, less than 20% attrition rate, and conflicts of interest on the part of the authors. The body of evidence from each study was evaluated using the suggested quality rating tools. The duration and results of the research were not restricted because BVN RFA is an innovative treatment.

Result

**Figure 1:**
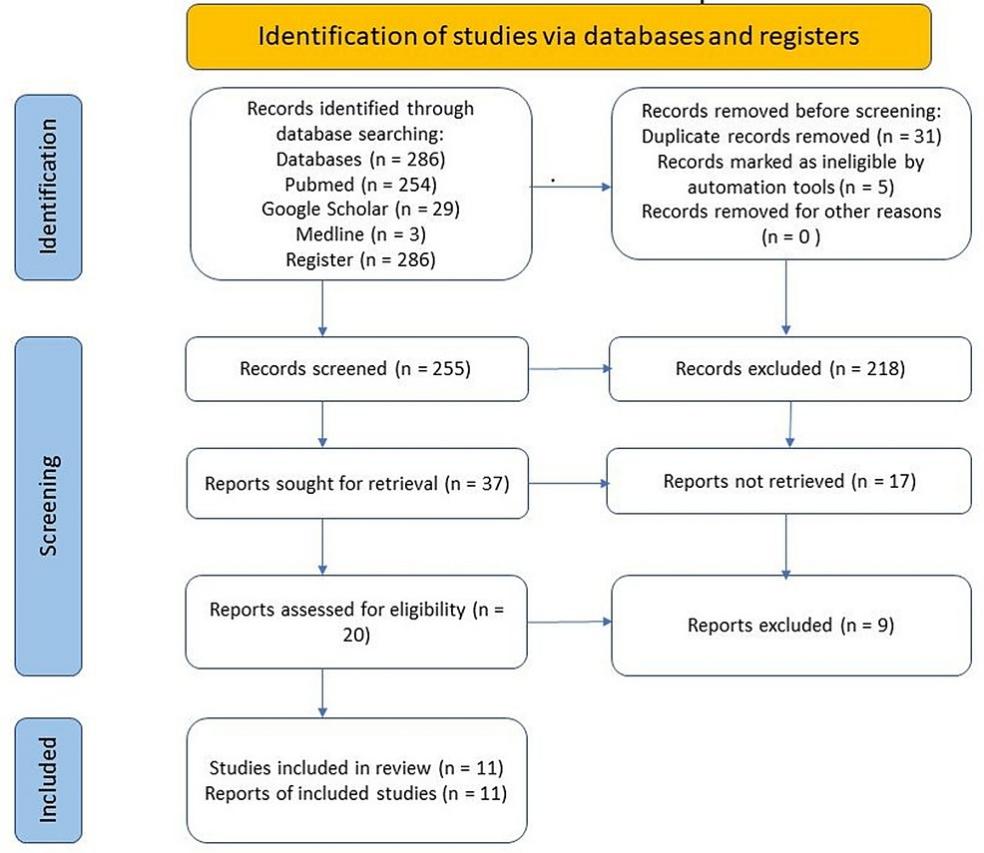
PRISMA Flow Diagram PRISMA - Preferred Reporting Items for Systematic Reviews and Meta-Analyses

The literature search and screening were conducted following the PRISMA guidelines, as shown in Figure 1 [23]. A total of 286 publications were extracted after the initial search. Following the removal of duplicates and irrelevant studies by abstract screening, a total of 11 studies were selected. Quality appraisal tools were used to assess individual studies for eligibility, and all 11 articles were included for review. After screening, the search generated one systematic review, one meta-analysis, three prospective randomized double-blinded studies, three prospective randomized open-label studies, one prospective single-arm, one randomized single-blinded, and one narrative review that met the criteria set for inclusion. The summary of the studies and study designs were organized in Table 2.

**Table 2:** Study Description and Quality Appraisal Result MeSH: Medical Subject Headings, SANRA: Scale for the Assessment of Narrative Review Articles, AMSTAR: A Measurement Tool to Assess systematic Reviews

Study Citation	Database	Year of Publication	Study Design	Quality Appraisal Tool Used	Quality Appraisal in Percentage
Conger et al. [2]	MeSH PubMed	2021	Systematic review	AMSTER	82.1%
Fischgrund et al. [3]	MeSH PubMed/Google Scholar	2018	Prospective Randomized Double-Blinded	Cochrane Risk Assessment	85.7%
Fischgrund et al. [4]	MeSH PubMed	2019	Prospective Randomized Double-Blinded	Cochrane Risk Assessment	100%
Fischgrund et al. [5]	MeSH PubMed	2020	Prospective Randomized Double-Blinded	Cochrane Risk Assessment	100%
Khalil et al. [17]	MeSH PubMed	2019	Prospective, Randomized Open-Label Multicenter	Cochrane Risk Assessment	71.4%
Koreckij et al. [21]	MeSH PubMed	2021	Prospective, Randomized, Open-Label, Single-Arm, Multicenter	Cochrane Risk Assessment	71.4%
Macadaeg et al. [7]	MeSH PubMed	2020	Prospective, Single-Arm	Newcastle Ottawa Tool	88.9%
Markman et al. [8]	MeSH PubMed	2020	Randomized Single-Blinded Sham-Control	Cochrane Risk Assessment	71.4%
Smuck et al. [16]	MeSH PubMed	2021	Prospective, Open-Label, Randomized Controlled Trial	Cochrane Risk Assessment	71.4%
Tieppo Francio [11]	MeSH PubMed	2021	Narrative Review	SANRA	91.7%
Conger et al. [1]	MeSH PubMed	2022	Single Arm Meta-Analysis	AMSTAR	93.75%

Prospective randomized control studies

In 2018, Fischgrund et al. published the result of a prospective multicenter randomized sham-controlled double-blinded clinical trial on 225 patients comparing BVN RFA to sham treatment [3]. The objective of the study was to evaluate the safety and efficacy of the BVN RFA for treating chronic vertebrogenic pain. The participants were skeletally mature adults with greater than or equal to six months of CLBP who had not responded to at least six months of conservative management and had MRI evidence of type 1 or type 2 MCs on one or more vertebral bodies from lumbar vertebra three through sacral vertebra one (L3-S1 vertebrae), a minimum ODI of 30 points, and a minimum VAS of four centimeter (cm), and treatment was minimized to two and a maximum of three consecutive vertebral bodies from L3-S1 vertebrae. Radicular pain, prior lumbar spine surgery, symptomatic spinal stenosis, osteoporosis, disc extrusion or protrusion greater than five millimeter (mm), spondylolisthesis greater than 2 mm at any level, three or more Waddell's signs of inorganic behavior, and a Beck Depression Inventory (BDI) score of greater than 24 were the exclusion criteria. Radicular pain is any pain radiating down the lower extremities in a dermatomal distribution, and symptomatic spinal stenosis presents with neurogenic claudication. The participants were 91% Caucasians with a mean age of 47 years; 17% were smokers, 59% were college-educated, and 74% worked before the procedure.

Patients were randomized 2:1 to either the treatment or the sham arm. Of the 225 patients, 147 were assigned to the treatment arm and 78 to the sham treatment, 113 received general anesthesia, and 112 received moderate conscious sedation. The patients in the treatment arm received transpedicular bipolar BVN RFA at 85 degrees Celsius (^o^C) for 15 minutes under image guidance targeting the terminus of the BVN at about 40% to 60% of the posterior to the anterior distance across the vertebral body. In the sham arm, the exact same procedure was followed, with the exception of docking the introducer cannula to stimulate radiofrequency ablation (RFA) for the same amount of time. At six weeks and six months, the patients were observed. The patients in the sham arm were allowed to cross over at one year. The ODI and the Medical Outcomes Trust Short-Form Health Survey (SF-36) were used to evaluate the disability and functional outcomes, and the VAS was used to assess back pain. The patients had MRI at six weeks and six months of follow-up to evaluate MCs. Both the patients and the physicians who conducted the follow-ups were blinded. The safety endpoints were the incidence and severity of adverse events (AEs).

The data analysis in the intention-to-treat (ITT) and per protocol populations was done in accordance with the FDA-approved protocol (PP). The targeting success was determined by the overlap of the RFA zone and the terminus of the BVN at the treated zone, evidenced on MRI at the six-week follow-up. Targeting success was observed in 89% (129 out of 145 patients) of the treated patients and 94.6% (300 of 317 vertebral bodies) of the treated vertebrae. The vertebrae treated were L3-S1 vertebrae. Targeting failure was observed in 16 patients; one was excluded for procedural failure, and two were excluded for protocol noncompliance in the RFA arm. Only one patient was excluded from the sham arm due to protocol noncompliance. In the RFA arm, the follow-ups at three, six, and 12 months were 98.7%, 98.2%, and 96.9%, respectively, and 98.7%, 97.4%, and 97.4% for the sham arm. Before the three-month evaluation, six patients exited the study, two withdrew their consent, three were removed after undergoing an invasive spinal procedure, and one died before the 12-month visit due to reasons unrelated to the study. At the three-month follow-up, the difference in ODI improvement between the two groups was statistically significant (p-value (p) less than (<).05), with 19.0 least squares mean (LSM) in the treatment group compared to 15.4 LSM in the sham group. The treatment group in the ITT and PP populations had 19.0 LSM and 20.5 LSM, respectively. The sham group had an ODI improvement of 20.5 LSM in the ITT group and 15.2 LSM in PP populations (p<.05).

However, the improvement in the ODI between the groups at six and 12 months was not statistically significant (p greater than (>).05). When a 10-point improvement in the ODI was utilized, 75.6% of patients in the treatment arm and 55.3% in the sham arm had a successful response. Referencing the target VAS of greater than 4 cm, the improvement in VAS among the two groups was not statistically significant at three months. The LSM improvement in VAS was 2.97 in the RFA group compared to 2.36 in the sham group (p >.05); the improvement in VAS at six and 12 months was statistically significant, 3.04 cm compared to 2.08 cm (p<.05) at six-month; and 2.84 cm in the RFA arm versus 2.08 cm in the sham arm (p<.05) at 12 months with a minimally clinically significant difference (MCID) for VAS of 1.5 cm. The change from baseline in the physical component of SF-36 in the PP for the RFA and the sham arm were not statistically significant at three, six, and 12 months. The MCID was 4.9. However, for the mental component of SF-36, the change from baseline in the PP group in both the treatment group and the sham arm was not significant at three and six months but was at 12 months (p<.05).

At one year, 73% (57 out of 78) of patients in the sham group were permitted to cross over to the RFA group, and data were collected only at three months. The high rate of crossover led to the conversion of the randomized clinical trial (RCT) to a nonblinded single arm intrapatient comparison in the BVN RFA arm at one year to minimize bias. AEs included one device-related event (sham group) and eight procedure-related events in six patients (two from the sham group). These AEs include nerve root injury in one patient in the sham arm, two cases of lumbar radiculopathy, retroperitoneal hemorrhage in one patient, and four transient motor or sensory deficits on the femoral nerve. The difference in the AEs between the two groups was insignificant, and all the cases were resolved. At six-week and six-month follow-ups, MRI did not demonstrate any spinal cord abnormalities, accelerated disc degeneration, or avascular necrosis except for a single case of a change from Modic type 1 to type 2. The researchers received industry funding.

In 2019, Fischgrund et al. published 24-month outcomes of the Sequential Multiple Assignment Randomized Trial (SMART) [4]. Among the 147 patients treated, only 106 patients from the BVN RFA arm completed a 24-month follow-up. They demonstrated a continued ODI mean improvement of 23.4 points (53.7%) compared to the 19.8 points improvement in one year (46.2%). The MCID was maintained at two years, with 76.4% (81/106) of the treated patients reporting continued improvement from BVN RFA at 24 months. On the 20-point threshold in ODI, 57.5% (61/106) of the treated patients showed improvement at two years. The mean VAS pain score reported at baseline was 6.73 cm, and the VAS improved by 2.76 (40.1%) at 12 months and 3.59 cm (52.9%) at 24 months from baseline.

The mean of the physical component summary (PCS) score of the SF-36 improved from 42.83 at 12 months to 45.83 at 24 months, demonstrating the effectiveness of BVN RFA in the treatment of vertebrogenic LBP. The analysis of the baseline characteristics of the 106 patients treated at 24 months demonstrated that 68 patients had LBP for over five years, 28 were on opioids, and 61 had prior spinal injections. However, at 24 months, only eight treated patients received spinal injections for continuing or new onset LBP. Opioid usage at two years was not assessed due to irregularity of visits on the part of the patients, but at 12 months, 60.7% of the patients had a decrease in opioid usage, and 46.4% stopped opioid use. At one year, two patients treated with RFA had surgical spine interventions, one had lumbar fusion at the treated levels, and one microdiscectomy at lumbar vertebrae two-three (L2-3) level above the indexed levels due to new disc herniation. Additional seven RFA patients had a lumbar fusion at the RFA levels. There were no reported late complications. The study was industry-funded.

In 2020, Fischgrund et al. published five-year outcomes on the SMART Trial targeting the 117 patients who were successfully treated out of the 133 patients that underwent the treatment in the per-protocol population from the original trial [5]. However, only 100 patients were available for follow-up at five years. At five years following BVN ablation, there was a 60.6% mean reduction in the ODI compared to baseline (p<.001), there was a 4.38 mean decrease in the VAS; 66% of the patients had a greater than 50% decrease in the VAS, 47% had a greater than 75% reduction in the VAS; and complete resolution of pain in 34% of the patients. At five years, 77% of patients had greater than or equal to 15 points improvement in the ODI, and 88% had greater than or equal to two-point improvement in the VAS (p<.05); eight patients were using opioids at least once a week compared to 30 patients at baseline, a 73% reduction. Only four patients received spinal injections in the prior year compared to 59 at baseline. Patient satisfaction was also improved, with 70% of the patients reporting a high degree of satisfaction, 27% had no change, 3% had worsening symptoms, and 65% of the patients reported resuming regular activities enjoyed before the onset of back pain. There were no reported late complications. The study was conducted with industry funding.

In 2020, Markman et al. published outcome data on a one-year post hoc analysis of the SMART Trial on opioid use [8]. In this analysis, data were collected from the 225 participants in the SMART Trial comparing the effectiveness of BVNA in patients on opioids. A week before each follow-up visit, data were collected on patient-reported opioid intake in addition to ODI, VAS, and SF-36 at baseline. The opioid used during the seven days was averaged and converted to MME. The threshold was set at 10 MME increase, decrease, or no change in opioid use. Opioid use below the 10 MME threshold was categorized as unchanged. At baseline, 50 out of 146 patients in the treatment arm and 27 out of 78 in the sham arm were on opioids. The baseline pain intensity was similar in both groups. At 12 months, patients who decreased opioid use had more remarkable improvement in the ODI and VAS and better pain relief than those with increased opioid use in the treatment and sham arms. After stratification, the mean opioid use in patients with increased opioid use rose from 12.1 at baseline to 14.8 and decreased from 15.4 to 4.0 (p<.001) in the reduced opioid group in the treatment arm. In the sham arm, the mean opioid use increased from 14 to 26 in the increased opioid use group and decreased from 24.2 to 3.9 (p<.001) in the decreased opioid use group at 12 months, respectively. The analysis was industry-funded.

Pragmatic studies

In 2019, Khalil et al. published the outcome of a multicenter prospective, randomized, open-label clinical trial on 140 patients with vertebrogenic CLBP comparing BVN ablation with standard care (the Intracept Trial) [17]. Among the 140 patients, only 104 completed the three-month follow-up; 51 patients were randomized to RFA arm and 53 to standard care (SC) arm. The participants were skeletally mature adults with greater than or equal to six months of CLBP. They had not responded to at least six months of conservative management, had MRI evidence of type 1 or type 2 MCs on one or more vertebral bodies from L3 through S1, a minimum ODI of 30 points, and a minimum VAS of 4 cm. Patients with radicular pain, symptomatic spinal stenosis, disc herniation greater than 5 mm, spondylolisthesis greater than 2 mm at any level, spondylolysis, MCs on levels other than L3-S1, and Beck Depression Inventory > 24, were excluded as well as those with prior lumbar spinal surgery, metabolic bone disease, prior fracture, spinal infection, facet arthrosis, on extended-release opioid with addiction behavior, BMI > 40, lumbar spine injury, bedbound, and contraindication to MRI. The demographics and baseline characteristics of the two groups were similar. The participants were 93% Caucasians with a mean age of 50 years; 9% were smokers, 50% were college-educated, and 78% were employed at the time of the trial. Only three-month results were reported; the clinical outcomes were assessed at baseline, three, six, nine, and 12 months using validated questionnaires.

For this study, the functional impact was measured with the ODI, the VAS was used for LBP, and SF-36 and European quality of life five dimensions (EQ-5D-5L) were used to measure Health Status and Quality of Life, respectively. The baseline ODI was 46.1, the VAS was 6.67, and the percentage of patients with CLBP for greater than five years was 67.3% and 70% had tried standard care. About 70% of the patients had received spinal injections and 16% had prior RF ablation of a facet or sacroiliac joint(s). RFA for BVN or SC was randomly assigned to each patient in a 1:1 ratio. The participants in the treatment arm received unilateral transpedicular intraosseous BVN RFA to any four vertebrae in a nonconsecutive approach for 15 minutes at 85^o^C under image guidance targeting the terminal of the BVN at approximately 30%-50% across the sagittal vertebral body width. In contrast, the patients in the standard care arm continued treatment with spinal injections, pain medications, exercise, physical therapy, acupuncture, and chiropractic management. The primary endpoint of the trial was set at three-month follow-up, and the prespecified provisional analysis for superiority assessment was conducted when 60% of the participants completed a three-month follow-up. The patients in the BVN RFA group were followed at six weeks then three, six, nine, 12, and 24 months postoperatively. SC patients were observed at three, six, nine, and 12 months. 

The primary outcome was a mean change in ODI, VAS, SF-36, and EQ-5D-5L scores from baseline. The study outcomes were analyzed with the intent to treat. At a three-month follow-up, the independent data management committee (DMC) recommended halting the enrollment in the study and offering patients in the standard care arm early crossover treatment to the BVN RFA arm due to the shown statistical superiority of the BVN RFA treatment over standard care on the prespecified outcomes (p<.001). The independent DMC also ruled out any safety concerns with the BVN RFA treatment. 

All 51 RFA patients completed treatment, 40 received two-vertebral bodies, and 11 received three vertebral bodies treatment, with lumbar vertebra five to sacral vertebra one (L5-S1) being the most frequently treated, followed by lumbar vertebra four to lumbar vertebra five (L4-L5) and lumbar vertebra four to sacral vertebra one (L4-S1). The mean duration of the procedure was 92.5 minutes. Patients in the standard arm received a spinal injection prior to the three-month endpoint, but none in the RFA arm. There was no significant difference in opioid reduction at three months among the two treatment groups. At the three-month follow-up, the RFA arm reported a 25.3-point (95% confidence interval (CI): -29.6 to -21.0; p<.001) decrease in the ODI from baseline compared to a 4.4-point (95% CI: -8.7 to -0.2; p<.001) decrease in the standard treatment arm. There was a 74.5% clinical success in the RFA arm compared to 32.7% of patients in the control group. In the RFA group, 62.7% reported greater or equal to 20 points improvement in the ODI compared to 13.5% in SC patients (p<.001). The RFA arm demonstrated a 3.46-point (95% CI: −4.10 to −2.82; p<.001) decrease in the VAS from baseline compared to a 1.02-point (95% CI: −1.66 to −0.37; p<.001) reduction in the standard arm; 2 cm improvement threshold was 72.5% of patients in the RFA arm compared with 34.0% of patients in the SC arm. 

The mental and physical components of SF-36 mental for RFA and SC were 2.615 (95% CI: 0.450-4.781; p<.001) versus −2.786 (95% CI: −4.952 to −0.620; p<.001); and 14.021 (95% CI: 11.995-16.048; p<.001) versus 2.114 (95% CI: 0.088-4.140; p<.001) respectively. The change from baseline in EQ-5D-5L was 0.1803 (95% CI: 0.1469-0.2137; p<.001) in the RFA arm compared with 0.0135 (95% CI: −0.0203-0.0472; p<.001) in the standard care arm, all favoring BVN RFA treatment. Among the 51 patients treated with RFA, 39 reported 78% improvement, eight had no change, and three patients reported worsened condition. The targeting success was observed in 96% (49 out of 51 patients) of the treated patients and 98% (111 of 113 vertebral bodies) of the treated vertebrae, and only two patients had target failure. Altogether, 22 AEs were observed; 15 were observed in 13 patients treated with RFA, including incisional pain, urinary retention, lateral femoral cutaneous neurapraxia, leg pain, and mild paresthesia, which resolved before the three-month follow-up. 

In 2021, Smuck et al. published the outcome of a prospective randomized multicenter open-label Trial on 140 patients with vertebrogenic CLBP comparing BVNA with standard care [16]. This trial is a 12-month follow-up outcome of the Intracept Trial. Of the 140 patients enrolled, 66 were randomized to BVNA and 74 patients to SC. The methods, inclusion and exclusion criteria, the primary and secondary outcomes, and the target success criteria were the same as in the Intracept Trial. In the end, 61 out of 66 patients were selected for BVNA, and five declined RFA treatment. At the time of enrollment, the mean age of the participants was 49.5; 80.3% of the patients had symptoms of LBP for greater than or equal to five years, the baseline ODI of 46.2; the VAS was 6.78; mean SF-36 PCS was 33.1; the mental component summary (MCS) was 49.2, and EQ-5D-5L was 0.61. At baseline, 23% of the patients were using opioids, 72% had received spinal injections, 8.2% had prior low back surgeries, seven withdrew in the SC arm for reasons not specified in the study, and one exited due to surgery for disc herniation. 

At six months, the difference in the reported outcomes was statistically significant, favoring the BVNA group. At the six-month follow-up, 67.2% of the patients reported a greater than or equal to 15-point reduction in ODI (p<.001) compared to 12.5% in the SC arm and a greater than or equal to 2 cm reduction in VAS in 58.3% of patients versus 6.0% in the SC arm (p<.001). There was no change in terms of chronic opioid use. At six months, 67.2% of the patients reported a greater than or equal to 15-point reduction in the ODI (p<.001) compared to 12.5% in the SC arm and a greater than or equal to 2 cm reduction in VAS in 58.3% of patients versus 6.0% in the SC arm (p<.001). There was no change in terms of chronic opioid use. At three and six months, the patients in the SC who were allowed to crossover to BVNA treatment had significant improvement in pain relief and function. There were a 24.7-point reduction in ODI from baseline at three months and a 25.9-point reduction at six months. The patients reported a 3.5 cm reduction in VAS at three months and a 3.8 cm decrease at six months. Among the treated patients, 65% reported a greater than or equal to 50% decrease in VAS, 36.2% reported a greater than 75% reduction, and 22.4% reported 100% pain relief six months post BVNA. Quality of life increased significantly from baseline in all patients treated with BVNA both at six and 12 months. At 12 months post-RFA, there was a 25.7-point mean reduction in ODI (p<0.001) and a 3.8-point reduction in VAS. In the BVNA arm, 64% of patients experienced a greater than or equal to 50% reduction in VAS, 44.3% attained a greater than 75% reduction, 29.5% of the patients achieved 100% pain relief, 68.9% had a greater than or equal to 15 points improvement in ODI, while 60.7% had a greater than or equal to 20 points improved ODI. The SF-36 and EQ-5D-5L improved significantly in one year.

Before the 12 months visit, 54.5% of patients in the BVNA arm received epidural steroid injections (ESI). At 12 months after BVNA, 4.5% received ESI of the same region versus 18.0% in the SC arm. Patients in the SC arm who received BVNA did not receive ESI at six months follow-up. At 12 months, 74% of patients in the BVNA arm reported improved pain relief, and 75% had treatment success. The patients in the SC arm who received BVNA reported 78% improvement and 72% treatment success at six months. Patient satisfaction and quality of life measured with SF-36 and EQ-5D-5L improved substantially. Following the 12-month follow-up visit, four patients reported postoperative leg pain secondary to the length of the procedure and positioning. Of the 127 patients treated with BVNA, 13 reported nonserious transient leg pain deemed device-procedure-related and resolved within 43 days. Other AEs included one case of urinary retention, one case of nausea, one case of skin rash due to prep solution, one case of corneal abrasion from surgical eye protection, and one case of infection to the incision site. No device-related severe AEs were reported during the 12-month visit. The study was industry-funded. 

In 2021, Koreckij et al. published the 24-month outcomes of the Intracept Trial [21]. In this trial, 140 patients were randomized, 66 patients to BVN RFA and 72 to SC. Among the 66 patients randomized to BVN RFA, 58 had a 24-month follow-up visit with an 88% retention rate; three patients were lost to follow-up. Among these patients, 67% reported greater than or equal to five years of LBP symptoms. The baseline mean ODI was 44.2, and the VAS was 6.6. The majority of patients had midline or paraspinal axial back pain, 22% had one or more BVNA treatments due to Pfirrmann grade III MCs on imaging; 50% of the patients had received ESI in the prior 24 months, 36% were actively on opioids, and 12% underwent previous low back surgery at the same level as the planned treatment within the last six months. Targeting success at the 24-month visit was 98% (130/132) of the treated vertebral bodies. At 24 months, patients in the treatment arm reported significant improvement in pain and function from baseline with a mean improvement in ODI of 28.5 and VAS of 4.1 from baseline of 44.5 and 6.6, respectively. In the BVNA arm, 72% of patients reported a greater than or equal to 50% decrease in VAS, 47% had greater than 75% reduction, and 31% had 100% pain resolution.

At two years, 77.2% of the patients reported a greater than or equal to 15-point reduction in the ODI (p<.001), a greater than or equal to 20-point reduction in 68.4% of patients (p<.005), and a greater than or equal to 2 cm reduction in VAS in 79% of patients. There were also significant improvements in SF-36 PSC and EQ-5D-5L. Following the 24-month follow-up visit, seven out of the 58 patients received ESI compared to 29 before the 24-month enrollment, a 76% reduction; three of the post-RFA ESI were at the BVNA treated levels. In the BVN RFA arm, 11 patients were on opioids at 24 months compared to 21 at baseline, a 62% reduction in active opioid use from baseline. In the BVNA arm, five patients out of 66 treated had spinal procedures performed at the treated levels at 24 months. Target success was observed in 72% of the BVNA patients without spinal injections post BVNA. At the two-year follow-up, in the BVNA arm, 79% of the treated patients reported improvement in the baseline condition, 21% had no change, 71% reported returning to enjoying pre-LBP activities, and 84% will repeat the procedure if needed. In the BVNA group, 11% reported nonserious device-procedure-related mild transient leg pain, and 12 out of the 13 AEs were related to pedicle breach. No device-related severe AEs were reported during the 24-month visit. The study was industry-funded. 

In 2020, Macadaeg et al. published the result of one-year outcome data of a prospective, single-arm study on 48 patients investigating the effectiveness of intraosseous BVNA for the treatment of LBP [7]. Of the 120 participants screened, only 48 patients were selected for the study. The participants were followed at six weeks and three, six, nine, and 12 months. Data from the 12-month outcomes were reported in this trial. The inclusion and exclusion criteria were similar to that of the SMART and the Intracept Trials. The primary endpoint was the improvement in the ODI, and LBP measured with change in the VAS; health status and quality of life were measured with SF-36 and EQ-5D-5L at three months post-BVN RFA, similar to the Intracept Trial. The targeting success was confirmed with an MRI performed six weeks post-BVNA. The electrode was positioned approximately 30%-50% across the sagittal vertebral body width from the posterior wall during the treatment utilizing the Intracept system technique. The unilateral transpedicular intraosseous BVN RFA was performed on any four vertebrae in a nonconsecutive approach for 15 minutes at 85^o^C under image guidance targeting the terminal of the BVN. Among the 48 patients treated, only 47 were successful. 

At the 12-month visit, only 45 patients followed up. The baseline ODI at 12 months was 47.13, the VAS was 6.82, 72.3% of the patients had a greater than or equal to five years of LBP, 75% were employed full-time, 21% had 2.5 days of work absenteeism due to LBP, 48.9% had received lumbar ESI, and 21.3% were on opioid. The targeting success was achieved in 96% of the treated patients and 98% of the treated vertebrae. The trial was performed on an intent-to-treat protocol. There was a significant improvement in pain and function from three months to 12 months post-BVN RFA. At 12 months post-BVN ablation, 89% of patients reported a greater than or equal to 15-point improvement in the ODI, and 84.44% reported a greater than or equal to 20-point improvement in the ODI from baseline (p < .001). There was remarkable improvement in the VAS, almost double the established MCID. At 12 months, more than 68% of the treated patients reported pain reduction greater than or equal to 50%; 51% of patients reported a greater than or equal to 75% decrease in pain, and 38% reported complete pain resolution. At 12 months post-BVNA, patients reported improved quality of life, as noted in the remarkable improvement in SF-36 and EQ-5D-5L. At 12 months post-BVNA, one patient had ESI for foraminal stenosis compared to 23 at baseline, one patient had facet ablation, and three patients were taking opioids compared to 10 at baseline, a 70% reduction. At one-year post-BVN ablation, 84.4% of the patients reported improved condition, 11% had no change, 4.0% had worsened symptoms, and only three missed work for two days. At 12 months, three nonserious device procedure-related events were reported; one was an aborted procedure due to inability to access the pedicle, and two were potential pedicle breaches resulting in radiculitis, which resolved after treatment with oral medications.

Meta-analysis

In 2022, Conger et al. published a single-arm meta-analysis on the effectiveness of intraosseous RFA of BVN in CLBP patients [1]. The meta-analysis was performed on patients greater than or equal to 18 years with CLBP and MRI evidence of Modic type 1 or 2 changes. The intervention of interest was intraosseous BVNA compared to sham, placebo, SC, or none. The primary outcome was a greater than or equal to 50% pain improvement in a VAS or NRS. The secondary outcome was an improvement in ODI by a greater than or equal to 15 points and a greater than or equal to 2-point improvement in NRS. Two databases, MEDLINE and Embase, were searched, 856 unique publications were screened, and 12 met eligibility criteria and were selected for review. The selected publications included both randomized and observational nonrandomized studies. The participants were 414 patients from a special population assigned to receive BVN RFA. Three independent reviewers evaluated the articles for appropriateness and eligibility, and the quality of evidence was assessed using the validated tools.

In this single-arm meta-analysis, the target success rate for 50% pain reduction at six and 12 months was 65% (95%, CI 51-78%) and 64% (95%, CI 43-82%), respectively. At six and 12 months, the ODI improvement rates of 15 points were 75% (95% CI 63-86%) and 75% (95% CI 63-85%), respectively. The authors also performed an ITT analysis of the success rate, which showed that 61% (95% CI 48-74%), 59% (95% CI 40-77%), 49% (95% CI 43-56%), and 50% (95% CI 41-58%) of participants reported a greater than or equal to 50% pain improvement at six, 12, 24, and 60 months, respectively. At six, 12, 24, and 60 months, the improvement in the ODI of a greater than or equal to 50-points was 71% (95% CI 59-82%), 70% (95% CI 57-81%), 57% (95% CI 50-64%), and 57% (95% CI 49-65%), respectively. The authors maintained that there is some evidence that BVNA reduces pain and disability in people with persistent LBP.

Systematic review

In 2021, Conger et al. published a systematic review (SR) of four publications assessing the quality of current evidence on the safety, efficacy, and complications of BVN RFA for CLBP [2]. Participants included adults greater than or equal to 18 years of age with CLBP and evidence of MCs on MRI. The intervention was intraosseous BVN RFA, and studies involving endoscopic extraosseous BVN RFA were excluded from the review. The comparisons were sham, placebo, active standard care, or none. The primary outcome was the percentage of patients with a greater than or equal to 50% reduction in pain in the VAS or NRS. The secondary outcomes included improvement in the ODI of a greater than or equal to 10-point, reduction in NRS of a greater than or equal to 2-points, mean changes in the ODI and VAS/NRS, and a decrease in the use of pain medication. There was no restriction on the duration of the outcome assessment. 

The studies selected for the review included RCTs, nonrandomized comparative studies, and single-group observational studies. The excluded studies were case reports, expert opinions, and manuscripts in a non-English language. The publications were obtained from MEDLINE and Embase. The search yielded 725 publications that were screened for duplicates and eligibility; seven studies were selected that met the inclusion criteria, and a total of four articles with detailed outcomes of 321 participants were reviewed. Two independent authors assessed the studies for inclusion using validated screening criteria, and the third independent author reviewed the studies for discrepancies. Studies were included if they presented information on the efficacy or effectiveness of intraosseous BVN radiofrequency neurotomy (RFN) for the management of CLBP, appropriate procedural information, rigorous study methodology, data analysis based on evidence-based medicine (EBM), and complications associated with BVN RFA.

The reviewers assessed the studies for methodologic rigor, risk of bias, and quality of evidence with the Grades of Recommendation, Assessment, Development, and Evaluation (GRADE) appraisal system. Seven publications were included in this systematic review. According to the authors of this SR, there is moderate-quality evidence supporting the effectiveness of BVN RFN for treating CLBP in patients with MCs in MRI using the GRADE measurement. Compared to standard care treatment or sham procedures, there is moderate-quality evidence that BVN RFN is more effective at lowering pain and impairment in carefully selected patients with CLBP. In patients with targeting failure, BVN RFN had minor to no measures of success over the sham procedure. The authors recommended high-quality non-industry-funded studies to confirm the outcomes of the studies.

Narrative review

In 2021, Tieppo et al. published the result of a narrative review on the effectiveness, safety, and rationale of basivertebral nerve ablation in the management of vertebrogenic LBP [11]. The databases searched included Medline, Pubmed, and Cochrane Library indexed manuscripts from the last 20 years. The search yielded 18 publications, five were eliminated for not meeting the inclusion criteria, and ten were selected for review. The eligibility criteria included studies on humans in English, randomized trials, meta-analyses, observational studies, and review articles. Two unblinded independent reviewers appraised the selected studies. Letters to the editors, book chapters, opinions, and case reports were all excluded. The use of the PRISMA diagram helped eliminate selection bias [23]. 

The authors concluded that BNV RFA is effective in managing patients with chronic vertebrogenic LBP lasting for at least six months with MCs type 1 or type 2 on MRI between L3-S1 spinal segments and who had failed conservative treatment for at least six months. Based on the reviewed articles, there was evidence that BVN RFA is more advantageous than standard care in treating CLBP. According to the authors, BVN RFA has a statistically significant benefit in pain reduction, quality of life improvement, opioid use reduction, and functional improvement. There were no severe adverse events, or device-related death observed. They concluded that BVN RFA is a safe and effective procedure for managing vertebrogenic pain using the correct procedural technique in an appropriate patient population. The generalizability of the study was a concern for the authors as the majority of the participants in the studies were Caucasians. There was an issue with all the studies being industry-funded. There was a recommendation to conduct a non-industry-funded research with a more representative patient population. This literature review was industry-funded. Table 3 summarizes all the studies used for this SR and the outcomes.

**Table 3:** Study Description and Summary of Results BVNA: Basivertebral nerve ablation, BVN: basivertebral nerve, SC: standard care, ODI: Oswestry disability index, VAS: visual analog scale, RFA: radiofrequency ablation, RFN: radiofrequency neurotomy, CI: confidence interval, CLBP: chronic low back pain, MCs: Modic changes, L3-S1 - lumbar vertebra three-sacral vertebra one

Authors and Year of Study	Study Design	Intervention	Control	Number of Participants	Outcomes
Fischgrund et al. [3]	Prospective Randomized Double-Blinded	Intraosseous Basi vertebral Nerve Ablation	Sham	225 patients. Randomization: 2:1. BVNA = 147 Sham = 78	The average ODI improved by 20.5 points in the treatment arm compared to 15.2 points in the sham arm (p = 0.019, PP) at 3 months. A responder rate based on ODI reduction 10 points revealed that 75.6% of patients in the treatment arm displayed a clinically significant improvement at 3 months, compared to 55.3% of patients in the sham control arm.
Fischgrund et al. [4]	Prospective randomized double-blinded	Intraosseous Basivertebral Nerve Ablation	Sham	147 BVNA = 128 Sham = 77	At 2 years, the average increase in ODI and VAS relative to the baseline was 53.7% and 52.9%, respectively. ODI 10-point improvement in 76.4% of patients and a 20-point improvement in 57.5% of patients; 70.2% had a 1.5 cm improvement in VAS.
Fischgrund et al. [5]	Prospective randomized double-blinded	Intraosseous Basi vertebral Nerve Ablation	Sham	117	At a 5-year follow-up, the average ODI score decreased by 25.95 points (Baseline= 16.86; at 5years = 42.81; p<0.001). The average reduction in VAS pain score was 4.38 points (Baseline = 6.74; p<0.001), 66% of patients had >50% pain reduction, 47% had >75% pain reduction, and 34% had complete pain remission. The composite responder rate utilizing thresholds >/=15 points on the ODI and >/=2 points on the VAS was 75%.
Markman et al. [8]	Randomized single-blinded	Intraosseous BVNA effects on opioid use	Sham	225 patients. Randomization: 2:1. BVNA = 147 Sham = 78	At 12 months, patients in the BVNA group with decreased opioid use had an ODI improvement of 24.9 ±16.0 versus 7.3± 9.0 (P 0.001) for those with increased opioid use. In the sham arm, the ODI improved to 17.4± 16.1 and 1.2 ±14.3 (P = 0.053) for patients with decreased and increasing opioid usage. Among the BVNA arm, in patients with decreased opioid usage, VAS improved to 3.3 ±2.5 (n = 27) compared to 0.6 ±1.8 (P 0.001) in patients with increased opioid use. In the sham arm, VAS improved to 2.5± 2.6 (n = 19) in the group with decreased opioid usage, compared to 1.4 ±1.9 (P = 0.374) in the group with increased opioid use.
Khalil et al. [17]	Prospective, randomized open label multicenter	Intraosseous BVNA	Standard care	140 patients Randomization: 1:1	At 3 months, the mean change in ODI for the BVNA group was 25.3 points, compared to 4.4 points for the standard care group (p<0.001). The mean increases in VAS for BVNA patients were 3.46 compared to 1.02 for standard care patients (p<0.001); 74.5% of BVNA patients had ODI 10-point improvement compared to 32.7% of standard care patients (p<0.001).
Koreckij et al. [21]	Prospective, randomized, open label, single-arm, Multicenter	Intraosseous BVNA	Standard care	140 patients BVNA = 66 SC = 74	At 2 years, the ODI improvement was 28.5±16.2 points (baseline = 44.5; p 0.001), and the VAS improvement was 4.1±2.7 cm (baseline = 6.6; p 0.001). Respond rates for ODI≥15 and VAS≥2cm were 73.7%. 72.4 % of patients achieved the target outcome of 50% pain reduction, and 31% experienced total pain remission. 5% of patients had steroid injections at the BVNA vertebral levels, while 62% continued to use opioids.
Macadaeg et al. [7]	Prospective, single arm	Intraosseous BVNA	None	48 patients	At 12 months, ODI improved by 32.31 ±14.07 (p<0.001); 88.89% had an ODI improvement of ≥15 points; VAS improved by 4.31±2.51 (p<0.001); >69% of patients achieved a 50% reduction in VAS. SF-36 and EQ-5D-5L scores improved by 26.27±17.19 and 0.22±0.15, respectively (p<0.001).
Smuck et al. [16]	Prospective, open label, randomized controlled trial	Intraosseous BVNA	Standard care	140 patients BVNA = 66 SC = 74	At 12 months, the mean ODI improvement in BVNA patients was 25.7±18.5 points (p<0.001); the mean VAS improvement was 3.82.7 cm (p<0.001); 64% of patients reported 50% pain reduction, and 29% reported complete pain relief. 92% of SC patients who transitioned to BVNA reported an improvement in ODI of 25.9±15.5 points (p<0.001). There was no difference in the percentage of opioid users between the two groups (p=0.56).
Conger et al. [1]	Meta-analysis	Intraosseous BVNA	Sham, placebo, standard care, or none	12 publications of 414 patients	intention-to-treat analysis for success rate, which showed that 61% (95% CI 48–74%), 59% (95% CI 40–77%), 49% (95% CI 43–56%), and 50% (95% CI 41–58%) of participants reported ≥50% pain improvement at 6, 12, 24, and 60 months respectively. At 6, 12, 24, and 60 months the improvement in ODI of ≥ 50-points were 71% (95% CI 59–82%), 70% (95% CI 57–81%), 57% (95% CI 50–64%), and 57% (95% CI 49–65%), respectively. There is moderate evidence that BVNA reduces pain and disability in people with persistent low back pain.
Conger et al. [2]	Systematic Review	Intraosseous BVNA	Sham, placebo, standard care, or none	4 publications of 321 patients	Compared to standard care treatment or sham procedures, there is moderate-quality evidence that BVN RFN is more effective at lowering pain and impairment in carefully selected patients with CLBP. In patients with targeting failure, BVN RFN had minor to no measures of success over the sham procedure.
Tieppo Francio et al. [11]	Narrative Review	Intraosseous BVNA	Standard care	10 publications	BVN RFA has a statistically significant benefit in pain reduction, quality of life improvement, opioid use reduction, and functional improvement. BVNA is superior to standard care in managing patients with chronic vertebrogenic low back pain lasting for at least six months with MCs type 1 or type 2 on MRI between L3-S1 spinal segments and who had failed conservative treatment for at least six months.

Discussion

This systematic review aimed to evaluate the quality of evidence of the current published studies on the effectiveness, efficacy, safety, and complications associated with intraosseous BVN RFA for treating CLBP. Following the criteria outlined in established guidelines, a total of 11 studies with detailed outcomes of 413 participants with CLBP were selected and reviewed [24]. The origin and mechanisms of CLBP were formally associated with disc degeneration and have been studied for years. Unlike other causes of the specific LBP, CLBP has recently been associated with disc and endplate degeneration leading to the release of inflammatory mediators and subsequent changes in endplate morphology [3-5,8].

To date, conservative and invasive procedures to manage CLBP with MCs have not demonstrated durable improvement in disability and function. These include pharmacological and non-pharmacological strategies, including surgery following the World Health Organization's stepladder approach to managing chronic pain [25]. Epidural steroid injections, whether interlaminar, caudal, or transforaminal, have been used in treating discogenic LBP but have not been effective in treating CLBP with MCs [26]. Medial branch RFA, which includes the docking of a large-bore needle in a continuous mode parallel to the medial branch nerve to interrupt a significant segment of the nerve, has proven effective and durable in the management of LBP secondary to facet arthropathy [26,27]. RFA can also be used for targeted tissue destruction and pain relief in vertebral metastases [9-11,28].

Transforaminal and interlaminar endoscopic lumbar RFA have also been used to treat discogenic pain in patients with degenerative intervertebral disc or spinal stenosis due to disc protrusion [29,30]. Current evidence has shown the considerable benefit of neuromodulators such as spinal cord stimulators in managing failed back surgery syndrome, radicular pain, and other neuropathic pain [26,27,31]. However, these interventions have not been influential in managing vertebrogenic pain with MCs. Prior studies have described transforaminal epiduroscopic basivertebral nerve laser ablation as promising in carefully selected patients with CLBP associated with MCs [29,30]. According to recent studies, BVN RFA has demonstrated durability and effectiveness in managing CLBP with MCs.

The BVN RFA was compared to sham treatment in the SMART trial and SC in the Intracept trial. These studies provided strong evidence supporting the vertebral endplate as the source of CLBP and BVN as a crucial player in transmitting pain signals in patients with CLBP. The studies evaluated in this review showed that individuals with axial persistent LBP of vertebral etiology, with MCs type 1 or type 2 on L3-S1 lumbar segments, who have failed conservative treatments for at least six months and satisfy other criteria, can benefit from BVN RFA. In all the studies, functional outcomes were measured with ODI, improvement in pain with VAS, and quality of life was evaluated using the short form SF-36 and the EQ-5D-5L questionnaire.

Other outcomes assessed were patient satisfaction and reduction in opioid utilization. The data from the studies showed that in patients in the BVN RFA arm, the mean ODI decreased by more than twice the MCID. The targeting success, described as an overlap between the ablation zone created by RFA and the terminus of the BVN at each treated level, ranged from 89% to 100% of the treated patients confirmed by post-ablation MRI results at six months [1-5]. Lack of targeting success at any one level automatically eliminates the patient from the ITT group. The placement of the electrode relative to the terminus of the BVN determines the success rate of the ablation. In the SMART trial, the electrode was placed between 40% and 60% of the posterior to the anterior distance across the vertebral body; however, placing the electrode proximal to the terminus of the BVN, approximately 30%−50% across the sagittal vertebral body width improved the target success as was the case in Intracept trial.

In the SMART trial, the two groups observed the same protocol. The sham surgical procedure involved docking the introducer cannula 1-2 mm into the pedicle to stimulate the RFA with an equivalent dwell time as the BVNA without turning on the RF device. At the three-month follow-up, the BVN RFN failed to outperform sham treatment, without a statistically significant difference in the ODI between the two groups. At an ODI of greater than or equal to (≥) 15-point improvement, the BVNA did not significantly outperform the sham procedure with 59% (95% CI: 51-68%) versus 49% (95% CI: 37-60%) reported outcomes, respectively. In interpreting these outcomes, it is crucial to note that the outcome of a surgical treatment is a cumulative effect of the critical surgical element, placebo effects, and non-specific effects, which may have played a role in this study [32]. Therefore, the slight differences in outcomes between the groups are not unusual and may be due to the placebo effect.

The authors failed to report the follow-up outcomes in the ODI between the two groups at six- and 12-month follow-ups. The prior systematic review conducted by Conger et al. obtained these results by contacting the authors [2]. BVN RFA is generally considered safe and minimally invasive, with a very low rate of AEs reported among all clinical studies. Similarly, the BVNA and sham arms had clinically significant pain reduction from baseline, but the difference between the groups is not clinically significant. At a 50% pain improvement threshold, the BVNA arm reported 46% (95% CI: 37- 54%) compared to 37% (95% CI: 26-48%) for the sham arm. The absolute pain reduction in the VAS, set at 1.5 cm from baseline reported in the BVNA patients, was 65% (95% CI: 57-74%) compared to 64% (95% CI: 53-75%) for sham. Using the analysis of covariance (ANOVA), slight statistically significant differences in mean pain scores from baseline were reported at six and 12 months.

Though at six and 12 months, there were improvements in the ODI in both groups, the difference between the groups was not significant. The mean improvement in the ODI at the 24 months and at five years follow-up of the SMART trial was 53.7% (p<.001) and 60.6% (p<.001), respectively. A sustained improvement was also observed in pain reduction and quality of life. The trials found that BVN RFN was a safe, durable treatment for vertebrogenic pain with MCs, showing maintained clinical benefits in the ODI for the treatment arm at two-year and five-year post-treatment follow-up.

In the study by Khalil et al., BVNA was compared to SC [17]. The SC in this study included but was not limited to pain medications, exercise, physical therapy, chiropractic treatment, acupuncture, and spinal injections. The majority of the patients had tried and failed to improve on spinal injections (70%), physical therapy (70%), and RFA of the facet and sacroiliac joints (16%). At 12 months, the inclusion criteria were expanded to include those with prior back surgery (12% of patients). Since the participants were randomized to the groups, they shared similar characteristics eliminating selection bias. In this study, the BVNA arm was superior and outperformed the SC group with an ODI of ≥10-point pain reduction, a 74.5% in the BVNA arm versus 32.7% in the sham arm. At ≥20-point pain reduction, the patients in the BVNA arm reported 62% versus 13% in the standard care. At 12 months [16] and 24 months [21] follow-ups, efficacy of the BVNA was maintained, with 74% (12 months) and 79% (24 months) of the patients reporting improvement in function with a 73.7% (p < .001) combined MCID function and pain responder rate. The BVNA was superior to SC in reducing the VAS and improving quality of life and function. 

However, the inadequate quantifiability of the standard treatment made it difficult to express what BVNA treatment outperformed and posed a challenge in interpreting the results of this study. Prior studies have shown that poor descriptions of the SC in an RCT may alter the interpretation of the outcomes [33]. But the use of usual care in the RCT was justified because experts recommended using standard care when trials involve interventions outside the usual care [34]. The unblinded nature of this trial may have affected the outcome due to the potential overrepresentation of the BVNA treatment on the side of the investigators and the possible Hawthorne effect on the part of the patients. According to the authors, the study was multicenter, conducted in 20 sites across the United States. One may argue the uniformity and reliability of the data collection method due to the unblinded nature of the study. The study was also based on the three-month result from the prior research.

A similar result was reported in the study conducted by Macadeg et al. in patients with similar characteristics as the SMART trial [7]. However, the electrode placement was comparable to the Intracept trial at approximately 30%−50% across the sagittal vertebral body width. The BVNA demonstrated robust benefit in treating chronic nonradiating axial LBP in this study. The three-month interim analysis showed statistically significant and clinically meaningful improvements in pain and function that persisted through 12 months post-treatment. The responder rate for ODI at 12 months was 88.9% (≥15-point) and 84.4% (≥ 20-point) improvement in the ODI from baseline (p<.001). There was also a clinically significant improvement in VAS, quality of life, and function. Nevertheless, the study was single arm without control, randomization, and blinding, which might create bias. Evidence has shown that randomization in a clinical trial is the most reliable method for evaluating the effectiveness of therapeutic interventions [35]. In clinical studies, there is also a risk of certain expectations influencing research outcomes, and blinding eliminates such bias, especially when the response criteria are subjective, as is the case in pain alleviation [36].

The one-year post hoc analysis of the SMART trial observed the effect of BVNA on opioid utilization as a secondary outcome. At 12 months, patients who decreased opioid use before BVNA had remarkable improvement in the ODI and VAS and better pain relief than those with increased opioid use in the treatment and sham arms (p<.001). These findings showed an association between reduction in opioid use and functional benefits from BVNA. At the five-year follow-up in the SMART trial, 34% of patients were using opiates at baseline, and 22% completely stopped opiates by the five-year follow-up. The study by Markman et al. is the only one that solely focused on the correlation of opiate use and BVNA with improvement in function and pain as a primary outcome [8]. The study by Fischgrund et al. demonstrated that 60% of opiate users reduced opiate use in the treatment arm at 12 months follow-up compared to a 40% increase in usage in those with increased opiate use [3]. At 12 months, there were significant differences in functional improvement between patients who reduced opiate use after BVN RFA (mean ODI reduction 24.9 plus or mins (±) 16.0) and those who increased opiate use after therapy (mean ODI reduction 7.3±9.0) (p<.001). There was no statistically significant difference in functional improvement between these two groups in the sham arm. Additionally, a decrease in the VAS correlates with a decrease in opioid utilization at a 12-month follow-up, but the reduction was not clinically significant (0.6mm). The results of the study by Markman et al. indicated that pain and functional improvements were more strongly correlated with BVN ablation treatment than with the increase in opiate use [8].

The single-arm meta-analysis by Conger et al. showed moderate evidence supporting the effectiveness of BVNA in treating vertebrogenic pain with clinically significant improvement in pain and function at six months (65%) and 12 months (75%) after BVNA [1]. The responder rates were remarkably stable at 24 and 60 months, with less than 5% variance in the proportions of VAS/ODI responders estimated with ANOVA. The strength of this review was evident in the study design and the protocol followed in conducting the review and reporting the data, which were in accordance with the quality guidelines for systematic reviews. The study by De Vivo et al. showed that percutaneous computed tomography (CT) scan-guided intra-osseous BVNA appeared to be an effective, fast, and safe technique with a 100% success rate and a mean operative time of 32 minutes [37]. It also showed a clinically significant success in improvement in the ODI (96.5%) and VAS (96.5%) at three months and 12 months.

Post-SMART studies have reported targeting success rates greater than 95%, proving that successful BVN neurotomy can be achieved frequently with established techniques [37]. Therefore, for BVNA to be effective, the precise target has to be achieved. In the SMART trial, the RFA target was between 40% and 60% of the posterior to the anterior distance across the vertebral body. However, there was a slight improvement in outcome when the electrode was placed approximately 30%−50% across the sagittal vertebral body width. Despite this, the targeting success was maintained at 90% in all the studies. As with RFA for facet arthropathy, precise electrode placement with fluoroscopic guidance is crucial in achieving success in BVNA [38]. Thus, adequate training is needed for proper technique to achieve successful targeting and neurotomy in BVNA to minimize complications.

The inclusion and exclusion criteria were based on the findings from prior research. The strictness of the inclusion and exclusion criteria made it challenging to generalize the research findings in this review. For instance, the inclusion of only patients with CLBP for greater than six months, isolated lumbar pain, lack of response to conservative treatment for at least six months (nonoperative management), and MCs Type 1 or Type 2 at L3-S1 levels. Additionally, a minimum ODI of 30 (on a scale of 100) and a minimum VAS of 4 cm (on a 10 cm scale) were required. In addition to the exclusion of patients with radicular pain, previous lumbar spine surgery, symptomatic spinal stenosis, osteoporosis (with T score 2.5), disc extrusion or protrusion greater than (>) 5 mm, spondylolisthesis > 2 mm at any level, and other spinal etiologies, patients with obesity and depression, and MCs at different levels, not L3-S1. It is worth noting that eligibility is crucial in any study because it protects the study participants and explains the target population for the study, but the extreme narrowness of the inclusion criteria can affect both the study result and generalizability [29,39]. However, given the novelty of this procedure, the restriction of the inclusion and exclusion criteria can be justified. On the other hand, in the Intracept trial by Khalil et al., the inclusion criteria were slightly liberal. The study included patients with microdiscectomy and laminectomy on extended-release opioids and moderate symptomatic spinal stenosis [17].

In terms of AEs, BVNA has proven to be safe. In all the studies, the AEs were relatively rare. The reported adverse events included nonserious and self-limited events such as nerve root injury (one patient), lumbar radiculopathy (two cases), retroperitoneal hemorrhage (one patient), and transient motor or sensory deficits on the femoral nerve (four patients). There were no spinal cord abnormalities, accelerated disc degeneration, or avascular necrosis on the post-BVNA MRI performed at six-week and six-month follow-ups. Other reported minor AEs included incisional pain, urinary retention, nausea, skin rash due to prep solution, transient leg pain, and mild paresthesia, which resolved before the three-month follow-up. There was one corneal abrasion from surgical eye protection, one case of infection at the incision site, and one aborted procedure due to the inability to access the pedicle with resultant transient radiculitis. No device-related severe AEs were reported during the 12-month visit. When BVN RFA becomes a routine procedure, other AEs and complications may be observed.

Also, large-scale studies may be essential in unveiling other AEs and complications not observed in these studies. BVN RFA remains an effective treatment for CLBP with Modic type 1 and type 2 changes. In the clinical studies, the independent data management committee recommended halting the enrollment and offered early crossover for patients in the control arm due to the compelling interim analysis result, which is unusual for the low back treatment study. The high success rate is attributable to selecting a well-defined subpopulation of patients with CLBP and a well-defined and precise technique. The randomization in the studies was successful, but the attrition observed was not significant (<1%); however, the authors did not explicitly report how it was accounted for during data analysis. It might affect the outcomes because attrition affects the ability to perform a full intention-to-treat analysis, which can lead to bias. In randomized trials, such attrition can substantially impact the strength of the research findings [40].

Another factor that may affect the generalizability of the studies was the demographic races of the participants. While the participants in Fischgrund (91.2%) and Khalil et al. (93.3%) were predominantly Caucasians [3,4,5,17], Macadaeg et al. [7] did not specify the proportion of different races in the study participants. Sadly, minority groups are continually underrepresented in clinical trials, even in modern clinical trials. It is problematic because treating a particular race with an intervention tried on another racial group in a clinical study may not yield the same outcome, and the efficacy and safety may differ in other racial subgroups [40]. Unfortunately, Hispanics and African Americans comprised 16% and 12% of the United States population, correspondingly. Despite this, the proportion of African Americans and Hispanics in the clinical trials is only 5% and 1%, respectively [41].

Heterogenous study participants improve the generalizability of clinical results and eliminate bias and skewness of the result of a clinical trial. Lack of diversity in clinical studies is not only a generalizability issue but also a moral, scientific, and medical issue. Thus, diversifying clinical trial participants is needed to help ensure that the trial population is representative of the patients who will use the medicinal product [41]. The authors could have done a better job recruiting participants from other racial groups. Though BVN RFA was superior to sham and standard treatment in the Caucasian population, the outcome in another racial group is unknown. Conducting a large-scale clinical trial on BVN RFA with diverse study participants is imperative.

It is worthwhile noting that the degenerative changes in the endplate noted in MRI were observed both in symptomatic and nonsymptomatic patients. Some patients with degenerative disc disease develop MCs while others do not. The pathophysiology behind this remains unclear. The review of a cross-sectional magnetic resonance imaging study showed that the endplate defects have a similar distribution pattern as MCs in the lumbar spine. Endplate defects were associated with MCs, decreased disc signal intensity, disc height loss, and increased disc bulging [42,43]. There is a strong association between Modic type 1 and 2 changes and chronic vertebrogenic pain in patients with CLBP unresponsive to conservative treatment [1-5,7-12,16,19,21,29,30,43]. Historically, discography was the gold standard for diagnosing discogenic pain, but it has become controversial due to high false positive rates. It is now known that the pain experienced by patients after provocative discography is due to endplate depression during the test.

Additionally, all the studies reviewed were industry-funded except the systematic review by Conger et al., increasing reporting and publication bias due to an inherent conflict of interest which may hinder the publication of negative results [1,2,44]. In industry-funded studies, there is a tendency to downplay the potential harms and emphasize the positive aspect of safety. Surprisingly, one review noted that trials funded by for-profit organizations had complete reporting of adverse events while trials funded by nonprofit organizations failed to report adverse effects. Despite this, the same review indicated that funding sources might impact the authors' interpretation and conclusions regarding the safety profile [44]. Though the comparisons of the results provided perspective on the strength of the study results, it does not conclude that BVNA is the superior treatment for all CLBP patients. Instead, it showed that it is very effective in the subgroup of chronic axial LBP with the outlined MRI criteria.

Many factors contributed to the strength of this review, such as the use of methodologic quality appraisal tools to evaluate the studies for inclusion. The review was designed and completed following the recommended quality and reporting guidelines unique to the systematic review. The review was executed following PRISMA and assessment of multiple systematic reviews-2 (AMSTAR-2) guidelines [1,2,23,45,46]. A comprehensive literature search, including the MeSH strategy, was used to extract relevant studies. The characteristics of the studies included were well defined, and study selection was performed in duplicate.

Limitations

The absence of grey literature is a limitation of the present review. Meta-analysis was not performed because of the novelty of the intervention and the scarcity of RCTs. The studies selected for the review were only free full-text studies with free access conducted in the United States and Canada within the last five years, retrieved from three databases MEDLINE, PubMed, and Google Scholar; all non-English language studies were excluded.

## Conclusions

BVN RFA is a safe and minimally invasive novel procedure for treating CLBP in patients with Modic type 1 and type 2 changes on L3-S1 vertebral levels. It is recommended in patients with CLBP lasting at least six months who have failed to respond to conservative treatment, including pharmacological and nonpharmacological therapies. The current studies have shown consistent evidence supporting the effectiveness and durability of BVN RFA in reducing pain and disability and improving function in CLBP patients for at least three months and longer. Evidence also proves that BVN RFA is superior to sham and standard care in managing CLBP patients.

The review provided evidence showing that BVN RFA has clinically significant benefits in a reduction in pain and disability, opioid reduction, and improvement in quality of life and function in patients with vertebrogenic pain with distinct Modic type 1 and 2 changes. The review also showed that BVN RFA is a safe procedure with few transient adverse events. There were no device-related deaths or serious AEs in reviewed studies. Additionally, nonindustry-funded large-scale, high-quality trials with generalizable study participants are needed to confirm the results of the current studies and further assess the AEs and complications. BVNA appears to be a safe and effective intervention for patients with vertebrogenic pain when proper patient selection and precise procedural techniques are employed.
